# Tuberculous Meningitis Associated with Diabetic Ketoacidosis

**DOI:** 10.4274/jcrpe.373

**Published:** 2011-12-06

**Authors:** Özlem Nalbantoğlu Elmas, Ayşehan Akıncı, Pelin Bilir

**Affiliations:** 1 Inönü University Faculty of Medicine, Department of Pediatric Endocrinology, Malatya, Turkey; 2 Ankara University Faculty of Medicine, Department of Pediatric Endocrinology, Ankara, Turkey; +90 422 341 06 60+90 532 643 53 50aakinci@inonu.edu.trInönü University Faculty of Medicine, Department of Pediatric Endocrinology, Malatya, Turkey

**Keywords:** diabetic ketoacidosis, type 1 diabetes mellitus, tuberculous meningitis

## Abstract

Diabetic ketoacidosis (DKA) is a life-threatening acute complication of type 1 diabetes mellitus.  Infections are the leading cause of DKA, but trauma, myocardial infarction, or surgery may also precipitate this condition. In patients with DKA, although cerebral edema is the most common cause of neurological symptoms, other possibilities such as meningitis or encephalitis should also  be considered. Herein, we present the case of an 8-year-old girl with DKA and tuberculous meningitis.

**Conflict of interest:**None declared.

## INTRODUCTION

Diabetic ketoacidosis (DKA) is a life-threatening acute complication of type 1 diabetes mellitus (T1DM). Infection, trauma, myocardial infarction, surgery are some of the conditions which lead to an increase in insulin requirements and thus to DKA (1). During the treatment of DKA, the patient’s neurological status may show impairment with symptoms of confusion, lethargy, stupor, or even coma.  Cerebral edema is the commonest cause of morbidity and mortality during the first day of treatment for DKA in pediatric patients (2). Although cerebral edema is the commonest cause of abnormal neurology in a child with DKA, other possibilities such as hemorrhage, thrombosis, and/or intracranial infection should also be considered.

## CASE REPORTS

An 8-year-old girl with T1DM was admitted to the hospital with fever, lethargy, anorexia, and vomiting.  She was diagnosed three years ago and had been on insulin therapy (four daily injections) since. According to the history obtained from her family, she had experienced nausea, vomiting, fever, lethargy, and headache for the last 24 hours. There was no history of previous neurological disease.  On physical examination, her temperature was 38.1�C, pulse rate was 112 beats/min, and respiratory rate was 30/min. She had Kussmaul respiration and was mildly dehydrated. Her history revealed no BCG vaccination. On neurological examination, lethargy and irritability were observed. There were no signs of meningeal irritation.

**Laboratory Findings**

On initial laboratory examination, the patient’s hemoglobin level was 15.5 g/dL. C-reactive protein (CRP) level was 5 mg/dL. Leukocyte count was 36 100/μL and differential count showed a shift to the left with toxic granulations in neutrophils. Blood glucose level was 444 mg/dL. Metabolic acidosis was present with a pH of 7.19. The other biochemical values were as follows: blood urea nitrogen level: 16 mg/dL, serum creatinine: 0.86 mg/dL, sodium: 124 mmol/L, potassium: 3.6 mmol/L, chloride: 102 mmol/L, serum osmolarity: 312 mOsm/kg. Hemoglobin  A1c was 8.1%. The patient’s urine was strongly positive for sugar and ketones.

**Management and Course**

**Figure 1:**
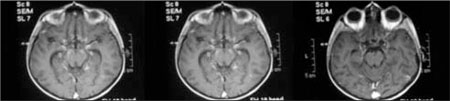
The tuberculous granulomas seen in the basal cisterna in contrast and non-contrast T1-weighted magnetic resonance imaging (MRI) in cross sections

**Figure 2:**
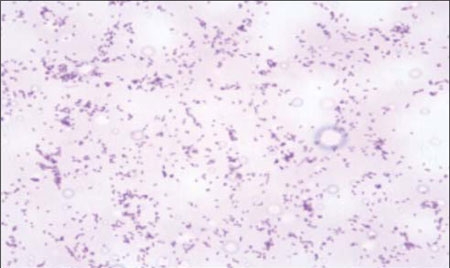
Acid- resistant bacilli demonstrated by cerebrospinal fluid staining

DKA treatment was started with intravenous fluids and insulin infusion. In view of the presence of fever, elevated CRP and elevated white blood cell count with neutrophilia, antibiotics were also started. Plasma glucose was monitored regularly. During the course of treatment, bradycardia developed and the lethargic state progressed to stupor.  Cranial computed tomography (CT) scan displayed cerebral edema. Lumbar puncture was not performed because of cerebral edema. Antiedema therapy was started with mannitol and dexamethasone. After 24 hours of treatment with intravenous insulin, blood glucose level decreased to 186 mg/dL. On the second day of treatment, diabetes insipidus occurred, and thus, desmopressin was added to the therapy.  With treatment, the metabolic parameters became normal, but the neurological status was not improved. Magnetic resonance imaging (MRI) was performed in order to detect any central nervous system (CNS) infection and revealed structures which were consistent with tuberculoma, such as is seen in tuberculous meningitis (Figure 1). After the resolution of the cerebral edema on repeat MRI, a lumbar puncture was performed. In the cerebrospinal fluid (CSF) examination, glucose level was 129 mg/dL, protein: 44 mg/dL, and leukocyte count was 3 ([i][/i]2 lymphocytes, 1 polymorphonuclear leukocyte). CSF staining demonstrated acid-fast bacilli, implying a diagnosis of tuberculosis (Figure 2). The patient was started on antituberculosis treatment (rifampicin, isoniazid, pyrazinamide, ethambutol). 

## DISCUSSION

DKA is a life-threatening acute complication of T1DM and if not diagnosed promptly and treated appropriately, may lead to further serious complications. DKA is commonly precipitated by inadequate insulin treatment or by an acute episode of infection. Infections were reported to be responsible for development of DKA in 32% of the cases. The infections which contribute to DKA include bacteria (49%), viruses (49%), and tuberculosis (2%) (3). Intracranial infections (meningitis/encephalitis) may be associated with DKA. DKA with tuberculous meningitis, herpes simplex type 2 encephalitis and group B streptococcal meningitis   have been reported (4,5,6).

Cerebral edema is the most common cause of acute neurological deterioration in DKA. However, in about 20% of acute neurological episodes, other causes such as localized edema due to infection,  hemorrhage or thromboses are detected by CT scan or on postmortem examination (7,8). Infection is also the most common precipitating factor for DKA and the symptoms of DKA such as fever, headache, confusion, irritability are typical findings in intracranial infections as well. Therefore, patients with fever, headache, confusion, aphasia, personality change, clouding consciousness and even coma should be further evaluated for intracranial infections (9).

The diagnosis of tuberculous meningitis is difficult in its early stages. Nonspecific symptoms seen in the early stage of this condition such as fever, headache, irritability, drowsiness are also common  in cerebral edema occurring in DKA patients. Therefore, in a type 1 diabetic patient, the symptoms of DKA could easily mask those of a concomitant stage I tuberculous meningitis, as in our patient. Our patient presented in a state of moderate ketoacidosis with fever, Kussmaul respiration and symptoms of dehydration.  Although metabolic control and blood glucose regulation were achieved through proper fluid replacement and insulin therapy, the neurological condition of the patient deteriorated, which was suggestive of brain edema or CNS infection. Suspicion for presence of CNS infection also increased due to continuation of high fever, leukocytosis and high CRP levels despite the improved hydration. The CT scan showed enlarged ventricles and tuberculomas, indicating tuberculous meningitis. Tuberculous meningitis is an infection that is caused by the hematogenous dissemination of tuberculosis bacilli from a primary focus.  In diabetic patients with poor management, immune resistance of the organism is low, which facilitates development and rapid dissemination of infections like tuberculosis. Therefore, particularly in countries with a relatively high incidence of tuberculosis, routine follow-up of diabetic patients should involve PPD tests and pulmonary radiographs.  

In conclusion, when the neurological condition of the patients with DKA deteriorates despite improved metabolic parameters, presence of concomitant CNS infections should be seriously considered. 
